# Physeal Sparing Posterior Cruciate Ligament Reconstruction in Paediatric Patients With Open Physes Using an Outside‐In Femoral Tunnel and a Low Tibial Tunnel

**DOI:** 10.1002/atn2.70239

**Published:** 2026-08-23

**Authors:** Shengkun Wu, Ruiying Pang, Ruoxi Zhang, Zhikuan Li, CongLei Dong, Jiangtao Dong

**Affiliations:** ^1^ The Third Hospital of Hebei Medical University Shijiazhuang Hebei China; ^2^ The Hebei Medical University Shijiazhuang Hebei China

## Abstract

In recent years, with increasing participation of adolescents in sports activities, the incidence of posterior cruciate ligament (PCL) injuries in paediatric patients has been increasingly reported. Conventional PCL reconstruction techniques typically require drilling femoral and tibial tunnels that cross or are adjacent to the physes. In paediatric patients with open physes, this technique carries a risk of damage to the physes, growth arrest, and lower limb deformity. For patients with open physes, a PCL reconstruction technique that both avoids physeal injury and restores knee stability is of critical importance. We describe a PCL reconstruction technique in this technical note. On the femoral side, a retrograde outside‐in drilling technique is employed to create the femoral tunnel distal to the physis. On the tibial side, using the meniscofemoral‐like structure as a reference, a low tibial tunnel is recreated.

## Graphical abstract

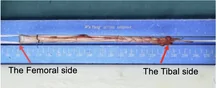

**VIDEO 1:** First, a routine arthroscopy is performed to examine the patient's anterior cruciate ligament, posterior cruciate ligament, and meniscus (side, left; position, “4”; anteromedial). When establishing the femoral tunnel, the center of the femoral tunnel aperture is positioned at the location where the anterolateral fibers of the posterior cruciate ligament remnant bundle are concentrated, approximately 5 mm distal to the cartilage margin (side, left; position, flex at 90°; anterolateral). Then, fluoroscopy is used to confirm that a drill bit of the same diameter as the graft will not damage the epiphysis. With the knee in the “figure‐4” position, a low posteromedial portal is established at the posteromedial soft spot to serve as the viewing portal. Under direct visualization, a high posteromedial portal is established 3 to 4 cm away from the low posteromedial portal as the operating portal. The posterior cruciate ligament guide is inserted beneath the meniscofemoral‐like structure (side, left; position, “4”; low posteromedial portal). Under fluoroscopy, it is ensured that a drill bit of the same diameter as the graft is positioned below the epiphysis. Under the action of the elevator, the adjustable loop titanium plate on the femoral side of the graft is pulled out from the outer aperture of the femoral tunnel along with the guidewire loop on the femoral side until the proximal marking of the graft on the femoral side completely enters the inner aperture of the femoral tunnel. With the knee in a neutral position and flexed at 90°, an “anterior drawer” test is performed to tighten the graft and ensure its tension. Video content can be viewed at https://doi.org/10.1002/atn2.70239.

Posterior cruciate ligament (PCL) injuries account for approximately 1% to 44% of all knee ligament injuries.^1^ In recent years, with increasing participation of adolescents in sports activities, the incidence of PCL injuries in paediatric patients has been increasingly reported.^2^ Conservative management of PCL injuries may result in residual knee instability and accelerated progression of medial compartment osteoarthritis. Therefore, for patients with open physes, a PCL reconstruction technique that both avoids physeal injury and restores knee stability is of critical importance.

Conventional PCL reconstruction techniques typically require drilling femoral and tibial tunnels that cross or are adjacent to the physes. In paediatric patients with open physes, this inevitably carries a risk of damage to the physis, growth arrest, and lower limb deformity.^3^ Consequently, restoring posterior knee stability and PCL function while minimizing physeal violation has become a central challenge in paediatric and adolescent sports medicine. This concern limits the applicability of traditional adult PCL reconstruction techniques in this population and highlights the need for safer and more precise surgical strategies.

We describe a PCL reconstruction technique for PCL injuries in paediatric patients with open physes. On the femoral side, a retrograde outside‐in drilling technique is employed to create the femoral tunnel distal to the physis. On the tibial side, the tunnel is created just proximal to the physis.

## SURGICAL TECHNIQUE

### Patient Positioning

The patient is in the supine position, and a thigh tourniquet is applied. A preoperative examination is performed under anesthesia (anterior drawer test, varus and valgus stress test, and pivot shift test).

### Graft Harvest and Preparation

**FIGURE 1:**
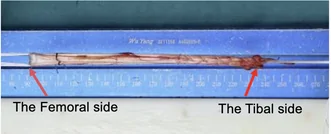
The final hamstring tendon graft.

An autologous hamstring tendon is selected as the graft. Excess muscle tissue and unstable portions of the graft are removed using a rasp. The graft is confirmed to have a diameter of 7 to 8.5 mm. The tibial end is whipstitched with FiberWire over a length of 3.0 cm. A marking suture loop is placed 20 mm proximal to the femoral end (Figure 1).

### Routine Arthroscopic Evaluation

**FIGURE 2:**
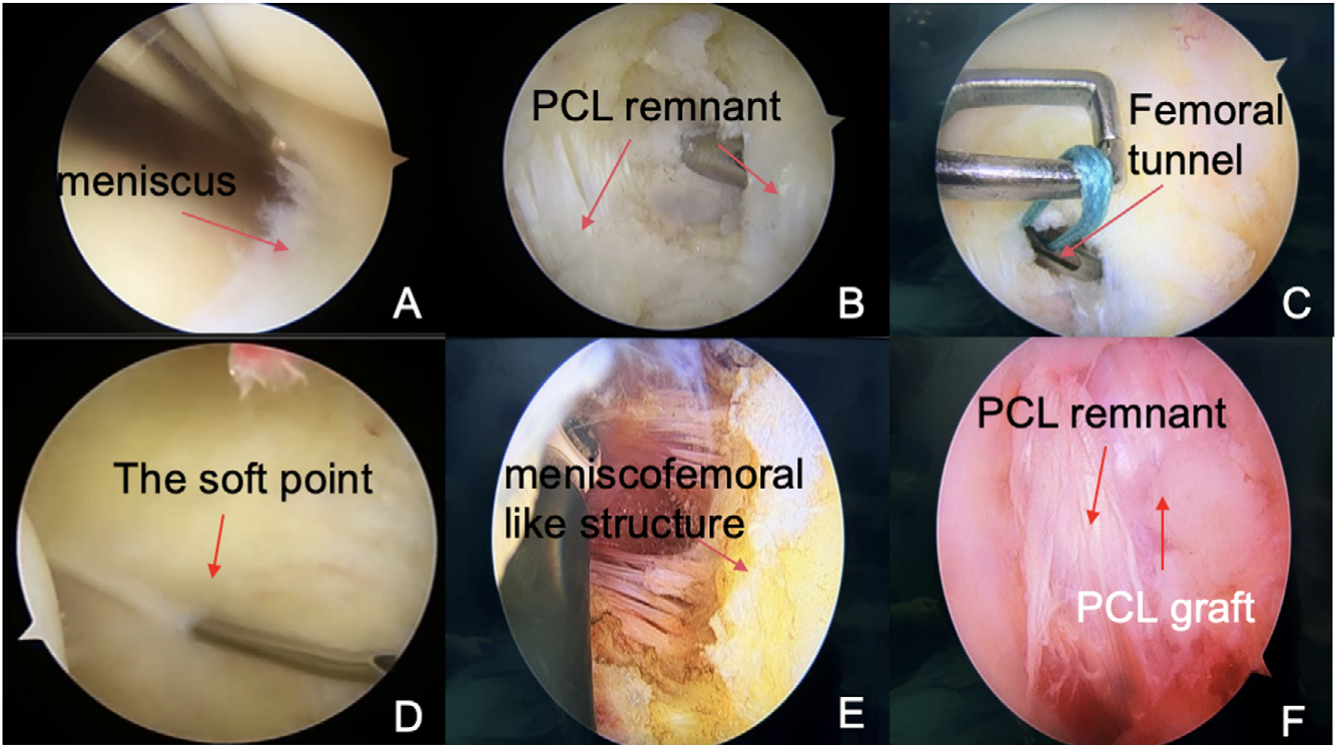
(A) Using a planner to explore the ACL, PCL, and meniscus (side, left; position, supine; anteromedial). (B) The center of the femoral tunnel is located at the site of the AL bundle approximately 5 mm distal to the cartilage (side, left; position “4”; anteromedial). (C) A guide pin is used to shuttle and retain a suture loop adjacent to the femoral tunnel (side, left; position “4”; anteromedial). (D) An LPM portal is established at the soft spot of the posteromedial side (side, left; position “4”; anteromedial). (E) The PCL tibial guide is positioned beneath the meniscofemoral‐like structure (side, left; position “4”; LPM). (F) The graft and PCL remnant (side, left; position supine; accessory anterocentral). (ACL, anterior cruciate ligament; LPM, low posteromedial; PCL, posterior cruciate ligament.)

First (Video 1), anteromedial (AM), anterolateral (AL), and accessory anterocentral portals are established to perform a routine arthroscopic evaluation including assessment of the anterior cruciate ligament, PCL and meniscus (Figure 2A).

### Creation of the Femoral Tunnel

**FIGURE 3:**
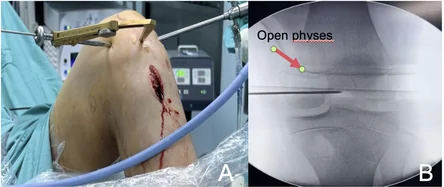
On the femoral side, the guide wire is inserted using an (A) outside‐in technique, and its (B) position is confirmed under fluoroscopy (side, left; frontal view).

An arthroscope (Conmed, New York) is inserted through the AL portal for visualization, whereas the AM portal is used as the working portal. The center of the femoral tunnel is located at the site of the AL bundle approximately 5 mm distal to the cartilage (Figure 2B). After the entry point is located, a guide pin is inserted horizontally from outside to inside followed by fluoroscopic confirmation that a drill of the same diameter as the graft will not violate the physes (Figure 3). A retrograde drill (Ruihe, China) is then used to create the femoral tunnel from outside to inside to approximately 15 mm. An orthopaedic planer introduced through an anterocentral portal is used to clean soft tissue to optimize the operative view, and a radiofrequency probe is employed to clear tissue around the tunnel for enhanced exposure. Try to preserve the PCL remnant as much as possible during the operation process. After femoral tunnel creation, a guide pin is used to shuttle and retain a suture loop adjacent to the femoral tunnel (Figure 2C).

### Creation of the Tibial Tunnel

**FIGURE 4:**
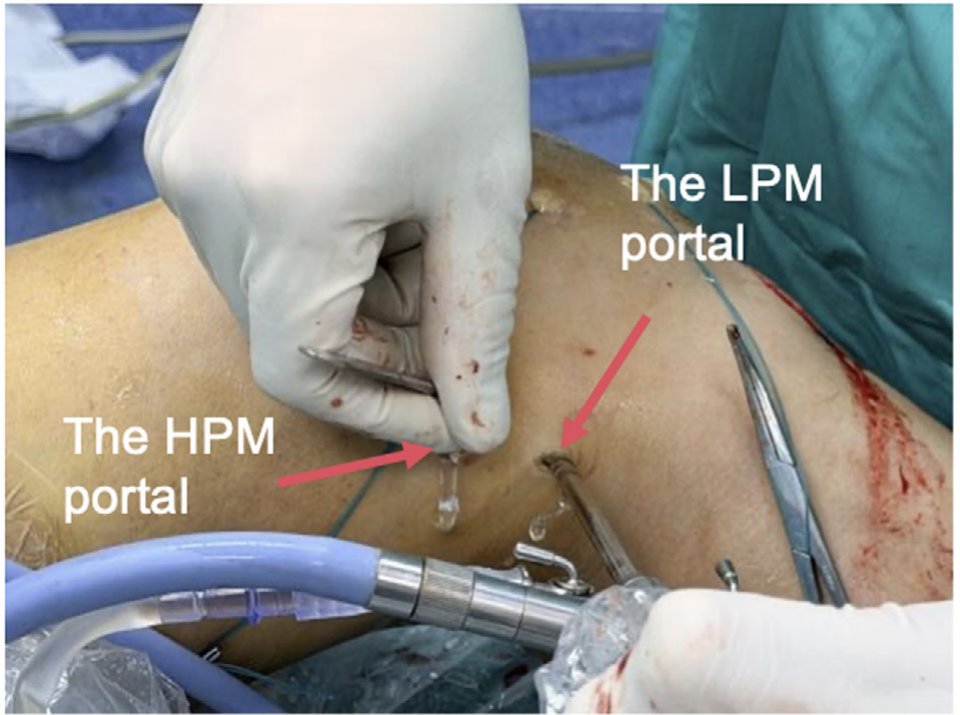
A HPM portal is created 3–4 cm proximal to the LPM portal. (side, left). (HPM, high posteromedial; LPM, low posteromedial.)

**FIGURE 5:**
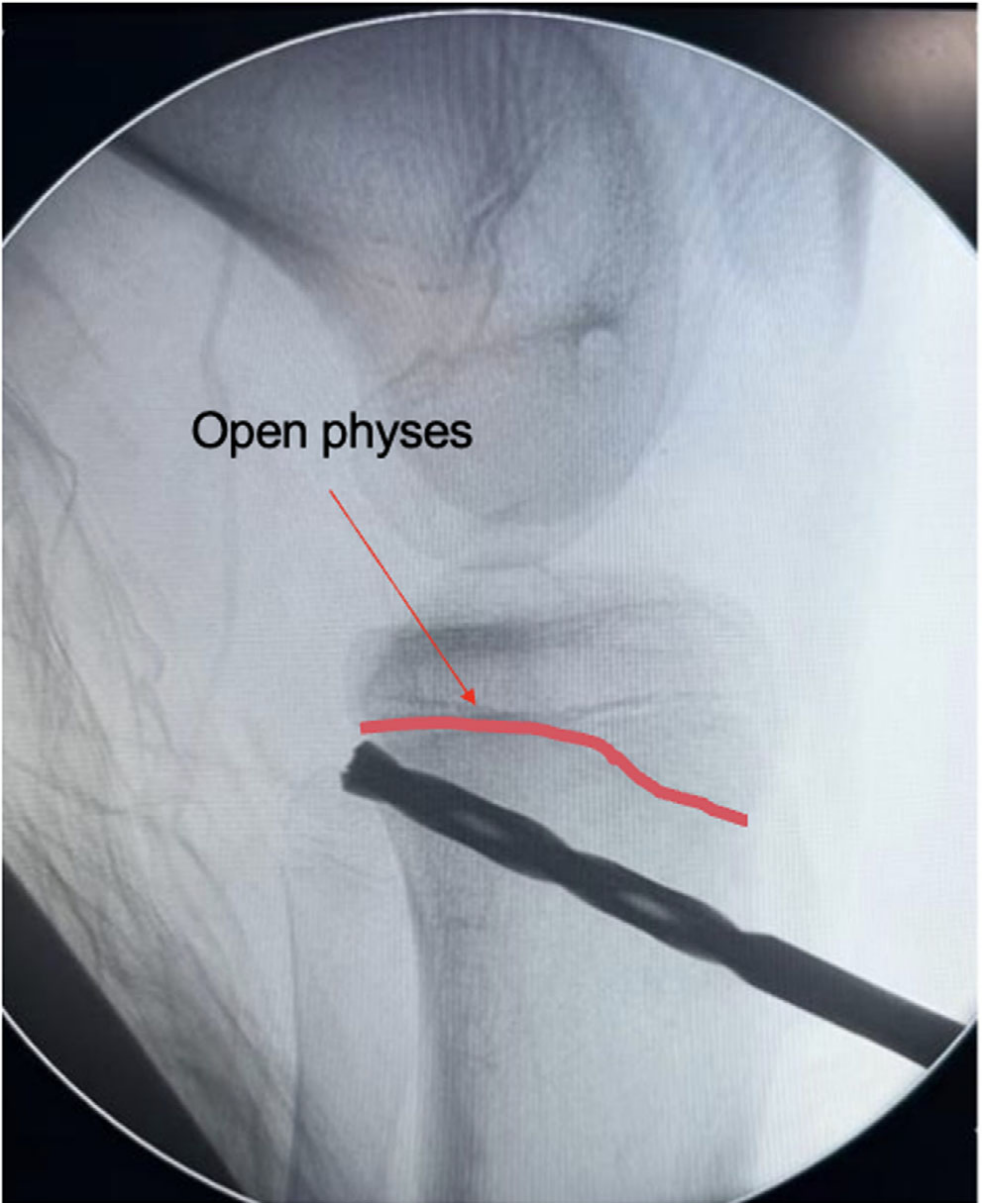
Under fluoroscopy, the tibial tunnel sized appropriately for the graft was created without damaging the physes (side, left; lateral view).

With the knee placed in “4” position, a low posteromedial (LPM) portal is established at the soft spot (near the medial head of the gastrocnemius) and used as the viewing portal (Figure 2D). Under direct arthroscopic visualization, a high posteromedial portal is created 3 to 4 cm proximal to the LPM portal to serve as the working portal (Figure 4). Soft tissue between the posterior capsule and the PCL remnant is cleaned while preserving the meniscofemoral‐like structure. The PCL tibial guide is positioned beneath the meniscofemoral‐like structure (Figure 2E), and an orthopaedic planer is used to retract the posterior capsule away from the operative field. A guide pin is then inserted from outside to inside through the medial side of the tibial tubercle. Under fluoroscopic guidance, it is confirmed that a drill corresponding to the graft diameter will remain distal to the physes (Figure 5). After creation of the tibial tunnel, a radiofrequency probe is introduced through the tibial tunnel to remove a small amount of surrounding tissue and ensure tunnel patency. During this process, avoid injury to the PCL remnant.

### Graft Placement

The graft is inserted through the tibial tunnel. Under visualization via the LPM portal, a tapered lever is inserted through the high posteromedial portal to lift the graft traction sutures, preventing entanglement with the PCL remnant. Assisted by the lever, the adjustable loop titanium button (Arthrex, Florida) on the femoral side of the graft, along with the femoral guidewire loop, is pulled out through the external orifice of the femoral tunnel until the proximal mark line of the graft fully enters the internal orifice of the femoral tunnel. Then, with the knee in neutral position and flexed at 90°, the anterior drawer test is performed to tension the graft, ensuring adequate graft tension (Figure 2F). Interference screw fixation is applied on the tibial side. After the 4 sutures from the tibial end of the graft are secured with the interference screw, they are passed through an external fixation button.

### Postoperative Care

After the surgery, the patient is required to wear a knee brace with posterior tibial support for 6 weeks to maintain knee extension. On the first postoperative day, the patient is encouraged to perform straight leg raises and ankle pump exercises. By the third postoperative week, the rehabilitation intensity is gradually increased. At 6 postoperative months, the patient is permitted to return to normal activities.

## DISCUSSION

During femoral tunnel preparation, we employed the outside‐in technique. Initially, the highest point of the femoral tunnel was determined lateral below the epiphyses. A guide pin was then driven from outside to inside in an inferior direction to create the femoral tunnel. This approach ensures that the entire femoral tunnel remains below the patient's epiphyses, thereby avoiding injury. For tibial tunnel preparation, the meniscofemoral‐like structure at the posterior aspect of the tibial plateau was used as a landmark to achieve as “low” a tibial tunnel position as possible. There is no unified standard for the positioning method of the lowest point. Studies have shown that a low tibial tunnel provides superior biomechanical performance in testing and helps reduce the adverse effects of the “killer turn” on the graft, thereby lowering the risk of graft failure.^4^

In this technique, we used 3 anterior portals: AM, AL, and anterocentral. Compared with the conventional 2‐portal (AM and AL) approach, the 3‐portal technique allows better visualization of both the femoral and tibial landmarks of the PCL, enhancing positioning accuracy. Posteromedially, we established 2 portals: a high posteromedial portal for instrumentation and an LPM portal for visualization. The dual posteromedial portals provide improved exposure of the posterior capsule and the tibial insertion of the PCL, helping to preserve the posterior septum of the knee and prevent injury. The posterior septum contains abundant vasculature, which may contribute to postoperative rehabilitation.^5^

The dual posterior approach increases the learning curve of the surgery, and it requires practice to fully expose the posterior field of view.

Ultimately, we preserved the residual posteromedial bundle of the PCL on the femoral side and reconstructed the PCL focusing on the center of the AL bundle. Preserving the PCL remnant serves 2 purposes: it may enhance graft revascularization and thus promote postoperative recovery, and it allows for a more anatomical reconstruction, ensuring the effectiveness of the surgical procedure. However, preserving the PCL remnant is disadvantageous for the surgeon to accurately locate the center of the bone tunnel. For beginners, when there is a conflict between disability preservation and precise positioning, we recommend focusing on accurate bone center.

Individualized PCL reconstruction does not impair growth in patients with open physes,^6^ yet no standard surgical technique exists for these cases. Our presented method offers a solution for patients. But our method still requires intraoperative fluoroscopy to ensure that the bone tunnel is not damaged by the epiphyseal plate. This PCL reconstruction procedure affects growth and requires further validation through long‐term follow‐up.

Table 1 lists the advantages and disadvantages, and Table 2 lists the pearls and pitfalls.

**TABLE 1:** Advantages and Disadvantages

Advantages
1. The femoral outside‐in technique better ensures that the femoral tunnel does not injure the epiphysis
2. Using the meniscofemoral‐like structure as a landmark allows creation of a low tibial tunnel, which reduces the adverse effect of the “killer turn” on the graft
3. Centering the femoral tunnel on the AL bundle of the PCL maximizes preservation of the PCL remnant, thereby promoting postoperative recovery

Disadvantages
1. Retaining the PCL remnant may obstruct the view and affect the positioning of the bone tunnel
2. For surgeons, the dual posterior approach requires a certain amount of learning time

AL, anterolateral; PCL, posterior cruciate ligament.

**TABLE 2:** Pearls and Pitfalls

Pearls
1. During debridement of the femoral tunnel, care should be taken to avoid damaging the PCL remnant
2. Retaining the PCL remnant may risk graft overstuffing. We therefore recommend using a graft diameter of 7 to 8 mm
3. Graft fixation should be performed while applying an anterior drawer maneuver to ensure proper graft tension

Pitfalls
1. There is no unified standard for the positioning of the low tibial tunnel
2. Intraoperative fluoroscopy is still needed to ensure that the patient's epiphysis is not damaged

PCL, posterior cruciate ligament.

## DISCLOSURES

The author (J.D.) declares the following financial interests/personal relationships which may be considered as potential competing interests: J.D. reports all support for the present manuscript (funding, provision of study materials, medical writing, article processing charges, etc.) from the National Natural Science Foundation of China, Hebei Medical University “14th 5‐Year Plan” Clinical Medicine Innovation Research Team Support Program, and Hebei Natural Science Foundation. The other authors (S.W., R.P., R.Z., Z.L., C.D.) declare that they have no known competing financial interests or personal relationships that could have appeared to influence the work reported in this article.
